# Mineralogical and Genomic Constraints on the Origin of Microbial Mn Oxide Formation in Complexed Microbial Community at the Terrestrial Hot Spring

**DOI:** 10.3390/life12060816

**Published:** 2022-05-30

**Authors:** Yuya Tsukamoto, Takeshi Kakegawa

**Affiliations:** 1 Atmosphere and Ocean Research Institute, The University of Tokyo, Chiba 277-8564, Japan; 2 Department of Earth Science, Tohoku University, Sendai 980-8574, Japan; kakegawa@tohoku.ac.jp

**Keywords:** hot spring, biogenic Mn oxides, multicopper oxidase, metagenome, anaerobes

## Abstract

Manganese (Mn) oxides are widespread on the surface environments of the modern Earth. The role of microbial activities in the formation of Mn oxides has been discussed for several decades. However, the mechanisms of microbial Mn oxidation, and its role in complex microbial communities in natural environments, remain uncertain. Here, we report the geochemical, mineralogical, and metagenomic evidence for biogenic Mn oxides, found in Japanese hot spring sinters. The low crystallinity of Mn oxides, and their spatial associations with organic matter, support the biogenic origin of Mn oxides. Specific multicopper oxidases (MCOs), which are considered Mn-oxidizing enzymes, were identified using metagenomic analyses. Nanoscale nuggets of copper sulfides were, also, discovered in the organic matter in Mn-rich sinters. A part of these copper sulfides most likely represents traces of MCOs, and this is the first report of traces of Mn-oxidizing enzyme in geological samples. Metagenomic analyses, surprisingly, indicated a close association of Mn oxides, not only in aerobic but also in anaerobic microbial communities. These new findings offer the unique and unified positions of Mn oxides, with roles that have not been ignored, to sustain anaerobic microbial communities in hot spring environments.

## 1. Introduction

### 1.1. General Background

Manganese (Mn) is ubiquitous in the Earth’s lithosphere and hydrosphere. Mn(II) is stable in solution under relatively acidic or anoxic conditions, whereas Mn(III) and Mn(IV) are favored under oxic or high pH conditions and, mainly, exist as Mn hydroxides, oxyhydroxides, or oxides [1]. The Mn cycling on the modern Earth is operated by shuttling between soluble Mn(II) and insoluble Mn(III) and Mn(IV). Mn(IV) oxides are found in diverse environments, including metal-contaminated streams [2,3], submarine hydrothermal fields [4,5], the ocean floor, where they occur as ferromanganese nodules and crusts [6,7,8], and terrestrial hot springs [9,10,11] (Table S1). Microbial Mn(II) oxidation is, generally, faster than abiotic Mn(II) oxidation processes [1]. This kinetic advantage implies that biological Mn(II) oxidation is thought to be widespread and significant, in natural environments over time [12,13,14]. However, little is known about the mechanisms of microbial Mn oxidation.

Direct mineralogical evidence of the biogenesity of Mn(IV) oxides is, still, obscure, due to the difficulties in distinguishing biogenic Mn(IV) oxides from abiotic Mn(IV) oxides. In contrast, several genetic pathways have been proposed for biogenic Mn(IV) oxides, including step-by-step enzymatic oxidation [15,16,17] and disproportionation of early biotic oxide [18]. Hence, more case studies of coupled examination of mineralogy, physiology, and enzymatic genomics, using natural samples, are required to, further, understand microbial Mn oxidation.

A total of 227 16S rRNA sequences of Mn-oxidizing bacteria have been reported from natural samples, through September 2021 (Table S2). It has become possible, in the last several years, to synthesize Mn (IV) oxides under environmentally relevant conditions, by incubating Mn(II)-oxidizing bacteria, and to compare their properties with those of synthetic Mn oxides [2,3,5]. Based on these experimental works, the understanding of the mechanisms of biological Mn oxidation has been well advanced. In addition, previous studies have discussed the benefits of biological Mn oxidation for Mn-oxidizing bacteria, in terms of protection from UV [19], oxidative stress [20], toxic heavy metals, reactive oxygen species, predators, and viral attack [21,22]. However, the significance of Mn oxidation in complex microbial communities is uncertain. Furthermore, Mn(IV) oxides have been the focus of researchers because of the chemical similarities between Mn oxides and the Mn-containing complex of photosystem II [23,24]. Therefore, studies on biogenic Mn oxides can, also, contribute to the understanding of the mechanisms of photosynthesis.

### 1.2. Mn Oxidizing Enzyme

Several enzymes involved in the biological Mn(II) oxidation and their essential roles have been previously described, e.g., [25,26,27,28,29,30]. Mn oxidases belong to two families of proteins in general: the animal heme peroxidases (AHPs) and the multicopper oxidases (MCOs). However, AHPs with Mn-oxidation capacity have been reported only in the marine bacteria *Erythrobacter* sp. SD-21, *Aurantimonas manganoxydans* SI85-9A1 [26], and *Roseobacter* AzwK-3b [29]. Recently, some algae have been confirmed to utilize extracellular proteins or superoxides for Mn oxidation [30]. MCOs include protein families, such as laccases, ferroxidases, and ascorbate oxidase. Crystal structures of more than ten MCOs have been determined, which have contributed to the understanding of their functions e.g., [31,32]. MCOs contain at least four copper (Cu) atoms that bind to specific amino acids. MCO particles were directly observed using transmission electron microscopy (TEM), and the size of the individual MCO particle was identified as 6 nm to 8 nm in diameter [33]. MCOs are prominent in all kingdoms and play a critical role in iron metabolism and copper homeostasis [34]. The activities of MCOs not only promote the oxidative metabolic cycle but also influence some diseases in animals [35]. In bacteria, some MCOs regulate the concentrations of Cu in cells, to avoid copper toxicity [36,37].

Previous experiments have demonstrated a link between genes encoding MCOs and Mn(II)-oxidizing enzymes [25,27,28]. In the past decades, Mn(II) oxidation experiments were performed using MCOs or putative Mn-oxidizing bacteria with MCOs (e.g., [25,26,27,28]). On the other hand, direct evidence of MCOs utilization is, still, missing for microbial Mn oxidation in natural environments.

### 1.3. Purpose of the Present Study

Mn(IV) oxides are actively precipitating in several terrestrial Mn-rich hot springs [38]. Meso to thermophilic Mn-oxidizing bacteria are believed to be involved in Mn(IV)-oxide formation. These Mn-rich hot springs are ideal for examining the physiology and enzymatic activities of Mn-oxidizing bacteria and their mineral products. In addition, Mn oxides occur deep in the hot spring microbial mat. This feature implies the certain role of Mn oxides (e.g., energy conservation) to sustain not only aerobic but also anaerobic microbial communities [39,40].

**Figure 1:**
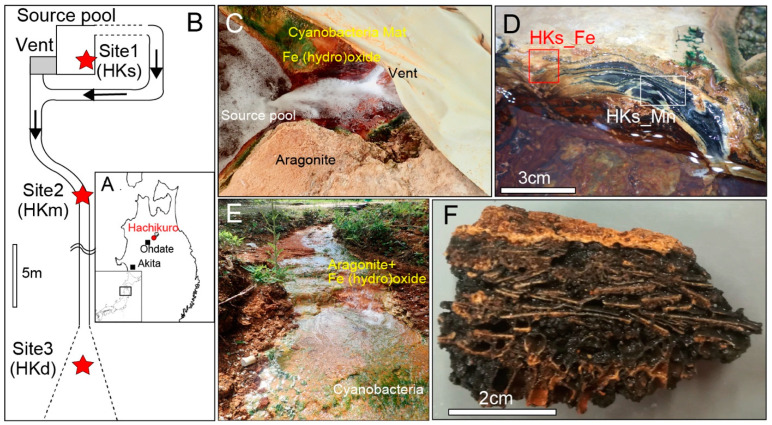
(**A**) Location of Hachikuro hot spring in Japan. (**B**) Relative locations of the sampling points in the hot spring stream. (**C**) Fe-(hydro)oxide sinters near the venting site. (**D**) Mn-oxide layers, beneath the cyanobacterial mat and aragonite layers at Site 1. (**E**) View of the mid-stream area (Site 2). (**F**) Mn-rich sample (HKm) at Site 2. Primary carbonate layers (orange part) are brecciated, with development of Mn oxides (black part).

Here, we present our novel genomic analyses, coupled with mineralogical and geochemical analyses, of the Mn-rich precipitates at an Fe- and bicarbonate-rich hot spring in Japan (Figure 1), to address the problems of the biogenesity of Mn oxides, enzymatic evidence of microbial Mn oxidation, and roles of Mn oxides to sustain hot spring microbial communities.

## 2. Materials and Methods

### 2.1. Site of Study

The Hachikuro (HK) hot spring is located in Akita, Japan (Figure 1A,B). Hot spring water is anoxic and rich in bicarbonate, at a source-venting area with moderate concentrations of Fe(II) and Mn(II) (Table S7). Ca-carbonate sinters of a mixture of aragonite and Fe-(hydro)oxides were deposited near the vent (Site 1, Figure 1C). The sinters were 2–5 cm thick and have laminated or columnar structures with a dark red color (sample HKs-Fe, Figure 1D). Some sinters were exposed to the air and covered by cyanobacterial mats. Black layers, confirmed as Mn oxides, commonly appeared beneath the cyanobacteria mats (e.g., sample HKs-Mn, Figure 1D). Fe-(hydro)oxides were less dominant in the downstream zones. Instead, the black- and brown-colored Mn-oxide layers became more significant. The hot spring water became slightly oxic, and the temperature decreased along the drainage path. The chemical composition, including Mn(II) concentration, did not change substantially (Table S7). Sinters at all sites were composed of Ca carbonates, but the chemistry changed from Fe-rich to Mn-rich, with increasing distance from the vent. Unsolidified carbonates were deposited at the midstream, approximately 10 m from the source (Site 2, sample HKm, Figure 1E,F). The thickness of the sediments was less than 5 cm, and they were covered by cyanobacterial mats. Black layers were, often, found beneath the cyanobacterial mat at this site. Sample HKd was a part of a Mn-rich soil that developed downstream, approximately 25 m from the source (Site 3). Sample HKd was brecciated and cemented with Mn oxides. Samples HKs-Mn, HKs-Fe, HKm, and HKd were used for further metagenomic, mineralogical, and geochemical analyses. After samples HKs-Mn, HKs-Fe, HKm, and HKd were retrieved, they were taken in sterile bags and, immediately, preserved in a freezer (−30 °C) in a laboratory. Weathered surfaces were removed to avoid contamination. Only the black part in the remnants were picked up and powdered, using a sterile mortar and pestle in a desktop clean bench for metagenomic and geochemical analyses.

### 2.2. Electron Microscope Observation

Micron-scale observations for the examined samples were performed, using field-emission scanning electron microscopy (FE-SEM, JSM-7001F, JEOL, Tokyo, Japan). Cross-sections of the alternating layers of Mn oxides and organic matter (Figure S1) were obtained, using a focused-ion beam (gallium source, FEI Versa 3D Dual Beam™, Thermo Fischer Scientific, Waltham, MA, USA). Nanoscale observations of the cross-sections were performed by TEM and scanning transmission electron microscopy (STEM), at an accelerating voltage of 200 keV (FEI Titan 3, G2 60-300 Double Corrector, Thermo Fischer Scientific, Waltham, MA, USA), which showed filamentous Mn oxides covered with organic matter. TEM (JEM-2100 and JEM-2010F, JEOL, Tokyo, Japan) observations were performed for powdered Mn oxides on the silicon grid, at an accelerating voltage of 200 keV. Measurement of the d-value of the diffraction pattern and its comparison with known minerals were performed using IPAnayzer (ver. 3.907) and RaciPro (ver. 4.806) [41].

### 2.3. Elemental and Stable Isotope Analyses

The elemental compositions of hot spring water at Site 1 and Site 3 as well as the trace elemental compositions of the Mn-rich soil (sample HKd) were measured, using inductively coupled plasma mass spectrometry (ICP-MS, Agilent 8800, Agilent, Santa Clara, CA, USA). The major elemental compositions of HKd were measured, using energy dispersive X-ray fluorescence (XRF, Epsilon 5, Panalytical, Almelo, The Netherlands). Concentrations of total carbon, total organic carbon, and total nitrogen were measured, using an elemental analyzer (Flash 2000, Thermo Fisher Scientific, Waltham, MA, USA). The total organic carbon content was determined, from the HCl-treated samples. Stable carbon and nitrogen isotope compositions were measured, using a Delta V Advantage Isotope Ratio Mass Spectrometer (EA-IRMS, Thermo Fisher Scientific, Waltham, MA, USA), through the ConFlowIV interface. The methods of the stable isotope analyses were followed by [42]. Briefly, approximately 15–30 µg of C and 50 µg of N were used for analyses. The isotopic ratios are reported, as δ^13^C values against the international standard Pee Dee Belemnite (PDB), δ^13^C = {(^13^C/^12^C)_sample_/(^13^C/^12^C)V_PDB_ − 1} × 1000, or as δ^15^N values against the international standard of atmospheric air, δ^15^N = {(^15^N/^14^N)_sample_/(^15^N/^14^N)_Air_ − 1} × 1000. The precision of the isotope analyses was confirmed, by repeated analyses of in-house reference material (histidine calibrated against IAEA reference materials), as ±0.2‰ (1 standard deviation).

### 2.4. DNA Extraction, 16S rRNA Gene Amplicon Sequencing, and Shotgun Metagenomic Analyses

#### 2.4.1. DNA Extraction and 16S rRNA Gene Amplicon Sequencing

After the samples were dried at room temperature, DNA extraction was conducted using the MoBio PowerSoil DNA Isolation kit (QIAGEN), in accordance with the protocol of the manufacturer, and eluted in 70 µL C6 solution. DNA concentration and quality were confirmed, using a NanoDrop (Thermo Fischer Scientific, Waltham, MA, USA) and QuantiFluor dsDNA system (Promega, Madison, WI, USA).

The microbial compositions were clarified by 16S rRNA amplicon paired-end sequencing, using the Illumina MiSeq platform (Illumina, San Diego, CA, USA). Sequencing was conducted at Seibustu–Giken Co. Ltd. (Kanagawa, Japan), and the V4 region (515F-806R) of the 16S rRNA gene was amplified by the bacterial primers 515F (5′-GTGCCAGCMGCCGCGGTAA-3′) and 806R (5′-GGACTACHVGGGTWTCTAAT-3′). Quantification of the 1st PCR products was confirmed, using the QuantiFluor dsDNA System. A 2nd PCR was conducted, using sequencing primers. Library concentrations and qualities were measured, using a Synergy H1 microplate reader (BioTek, Agilent, Santa Clara, CA, USA) with a QuantiFluor dsDNA System and on a Fragment Analyzer (Agilent, Santa Clara, CA, USA) with a dsDNA 915 Reagent Kit (Agilent, Santa Clara, CA, USA), respectively, in accordance with the instructions of the manufacturer. Paired-end sequencing (2 × 150 bp) was performed on the Illumina MiSeq platform (Illumina, San Diego, CA, USA), with an MiSeq Reagent Kit v3 (Illumina, San Diego, CA, USA). All library preparation, pooling, quality controls, and sequencing were conducted at the Seibustu–Giken Co. Ltd.

Reads beginning with a sequence that completely matched the used primer were extracted, by using the FASTQ barcode splitter tool within the FASTx-Toolkit (ver. 0.0.14). Trimming the primer sequence, removing chimeric sequences, and denoising were conducted using the QIIME2 pipeline (ver. 2020.11) [43]. Using the feature classifier in QIIME2, filtered and chimaera-free sequences were aligned and clustered into operational taxonomic units (OTUs), at a >97% similarity level against the SILVA138 database (Table S3) [44]. 16S rRNA gene data of known Mn-oxidizing bacteria were compiled from previous studies [2,3,4,8,9,10,25,26,39,45,46,47,48,49,50,51,52,53,54,55,56,57,58,59,60,61,62,63,64,65,66,67,68,69] (Table S2).

#### 2.4.2. Shotgun Metagenomic Analyses

To construct metagenomic assembled genomes (MAGs) of putative Mn-oxidizing bacteria, shotgun metagenomic analyses were conducted. After extracting DNA as well as confirming the concentration and quality of DNA, using the above protocol, DNA extractants were sent to Genome–Lead Co. Ltd. (Kagawa, Japan) for library preparation, quality control, and sequencing. Sequencing was performed, using 2 × 150 bp paired-end sequencing using DNBSEQ (BGI, Shenzhen, China). Shotgun metagenomic sequencing produced 11.04, 20.76, and 20.96 gigabases from the samples of HKs-Mn, HKm, and HKd, respectively. Assembly of raw sequences, binning, and refinement were performed, using MetaWRAP (ver. 1.2.1) [70]. Raw sequences obtained from each sample were assembled, using Megahit (ver. 1.1.3) [71] with default parameters, and contigs of less than 2000 bp were removed. Binning was performed using MetaBat2 (ver. 2.12.1) [72], MaxBin2 (ver. 2.2.6) [73], and CONCOCT (ver. 1.0) [74]. Bin refinements were performed on the bin refinement module in MetaWRAP, to obtain the final bin set. The completeness and contamination of the genome bins were assessed, using CheckM (ver. 1.1.3) [75]. A total of 114 MAGs, 45 MAGs, and 39 MAGs were obtained, respectively (Table S4) [76]. The taxonomy of each bin was assigned using the GTDB (ver. 1.4.1) [77,78,79]. Protein-coding gene prediction and translation to amino acid sequences were performed, using Prodigal (ver. 2.6.3) [80] with -p meta.

Putative Mn-oxidizing genes were extracted, based on four criteria: (1) >30% homology with known Mn-oxidizing genes; (2) compatibility with domain; (3) preservation of amino acid sequences of the four conserved copper-binding sites of MCOs [28,81], T1, T2, T3a, and T3b sites (Figure S2); and (4) location in the same clade with the assigned known Mn-oxidizing gene in the phylogenetic tree. The reference database of known Mn-oxidizing genes compiled in this study was screened against the concatenated protein FASTA sequences from MAGs, using BLASTP and HMMER ver. 3.3.2 (e-value cut-off of 10^−5^ against Pfam-A database), to extract putative Mn-oxidizing genes and confirm their domain. Afterwards, possessing four conserved-copper-binding sites of MCOs and the phylogenetic relationship tree between the queried sequences and assigned, the known Mn-oxidizing genes were confirmed. Alignment was performed, using MAFFT (ver. 7) [82] with default parameters. After removing the suspicious or poorly aligned regions, using trimAI (ver. 1.4) [83], phylogenic trees were constructed, using RaxML (ver. 8.2.12) [84] with the command “raxmlHPC-PTHREADS -m PROTGAMMAAUTO -p 12345 -x 12345 -T 8 -N 100”. MAGs possessing putative Mn-oxidizing genes, which met all the criteria, are listed in Table S5. References for the Mn-oxidizing genes used in this study are listed in Table S6.

Potential metabolic pathways were evaluated, using module completion ratios (MCR) and Q-value on Genomaple^TM^ (ver. 2.3.2) [85]. Following the official manual, functional modules with a Q value < 0.5 were adopted, to consider the metabolic pathways of queried MAGs. Gene annotation was performed, using DFAST [86,87].

## 3. Results and Discussion

### 3.1. Biogenic Mn Oxides in Fe- and CO_2_-Rich Hot Spring

**Figure 2:**
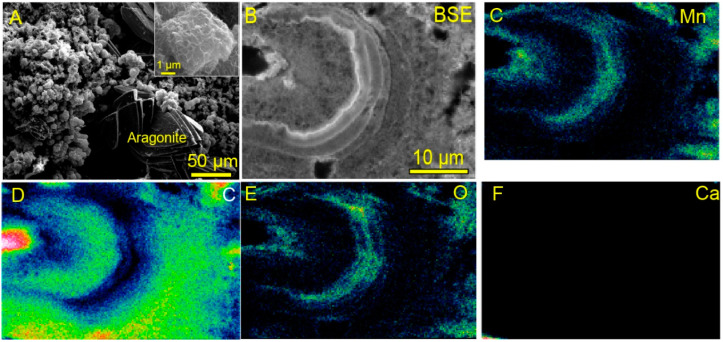
SEM image of Mn oxides and elemental distribution. (**A**) Well-faceted crystals represent aragonite and submicron spheres represent Mn oxides, in this image. (**B**) Back-scattered image of the cross section of a Mn-oxide sphere. Mn-oxide spheres show concentric inside structures, made of chemically distinct layers. (**C**–**F**) The distribution of Mn, C, O, and Ca, respectively. Bright layers in the back-scattered image (**B**) corresponds to MnO_2_ layers (**C**,**E**). Dark layers are made of organic matter, which was suggested because Ca (**F**) and O (**E**) were not detected.

**Figure 3:**
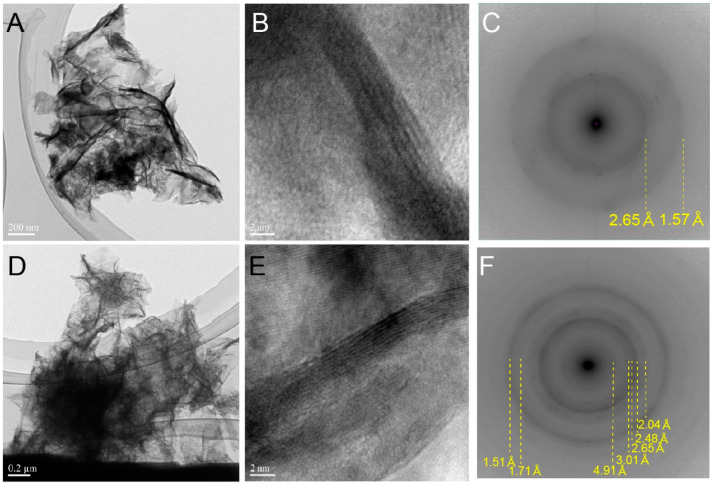
HR-TEM images of Mn oxides. (**A**) TEM image, (**B**) lattice image, and (**C**) diffraction pattern (HAADF) of Mn oxides in HKs-Mn. Based on HAADF, this crystal is identified as δ-MnO_2_ (vernadite). (**D**) TEM image, (**E**) lattice image, and (**F**) diffraction pattern (HAADF) of Mn oxides in HKm. Based on HAADF, this crystal is identified as hausmannaite (Mn^2+^Mn^3+^_2_O_4_). (**A**,**D**) do not show clear crystal structures. A part of lattices of Mn oxides are captured in (**B**,**E**), but most parts do not show clear lattices. HAADF images of both samples are ringed, suggesting X-ray diffraction from multiple weakly crystalline crystals.

Assembled spherical Mn oxides (<5 µm in diameter, Figure 2A) in each sample were observed by FE-SEM. The spheres consisted of alternating layers of Mn oxides and organic matter (Figure 2B–F). Such a spatial relationship between organic matter and Mn oxides is unique and has, rarely, been reported in modern marine Mn nodules or crusts. Organic matter is encrusted by linear, folded, and fibrous forms of Mn oxides, at the nanoscale (Figure 3A,B,D,E and Figure S1, HKs-Mn, HKm, and HKd). Most Mn oxides in the examined samples were amorphous in phases, but some of the Mn oxides showed randomly stacked lattices in the TEM images (Figure 3B,E). High-angle annular dark field scanning (HAADF) TEM analyses revealed that the examined samples were composed of poorly crystalline phyllomanganates. δ-MnO_2_ (vernadite, Figure 3C), hausmannite (Mn^2+^Mn^3+^_2_O_4_, Figure 3F), and birnessite ((Na,Ca,K) × (Mn^4+^, Mn^3+^_2_)_2_O_4_·1.5H_2_O) were identified in the examined samples. TEM images (Figure 3A,B,D,E and Figure S1) are, apparently, different from synthetic triclinic MnO_2_ [88]. The biogenesity and genetic sequences from vernadite, hausmannite, and birnessite have been discussed, by previous investigators [12,89,90]. Incubation experiments of Mn-oxidizing bacteria, also, produced poorly crystalline birnessite [39]. Our observations are consistent with the previously proposed biogenic origin models of these phyllomanganates [12,89,90,91].

Previous studies have reported the enzymatic or biogenic formation of Mn-oxide nanoparticles as a nascent phase [33,92]. However, nanoparticles of Mn oxides were not found in the examined samples, suggesting early rapid merging of nanocrystals into larger polycrystals, in the hot spring environments. Tunnel-structured manganates, such as todorokite, are common in Mn nodules on the modern ocean floor [93,94,95], but these were not present in the examined samples in the present study.

### 3.2. Complex Microbial Community at HK

**Figure 4:**
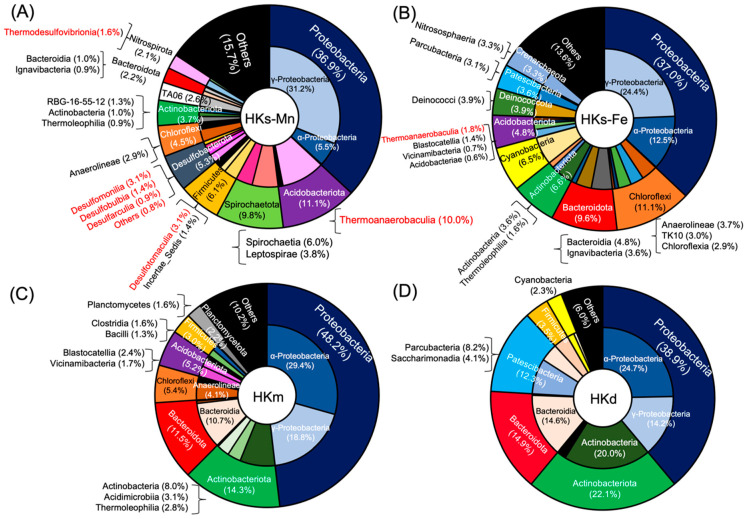
Community structure based on 16S rRNA of (**A**) HKs-Mn, (**B**) HKs-Fe, (**C**) HKm, and (**D**) HKd. Red color indicates anaerobes. The outer pie chart represents phylum level and the inner pie chart represents class level.

Metagenomic analyses for biogenic Mn oxides indicated different phylogenies at each sampling point. The phylum- and class-level community compositions are illustrated in Figure 4 and Table S3. One-third of the operational taxonomic units (OTUs) at each site were, generally, composed of members of *Gammaproteobacteria* and *Alphaproteobacteria*. The abundance of Proteobacteria was the same in HKs-Mn and HKs-Fe.

*Actinobacteriota* and *Bacteroidota* are abundant in HKm and HKd, compared to HKs-Mn and HKs-Fe. *Acidobacteriota* and *Chloroflexi* were more abundant in HKm than in HKd. OTUs of *Pastescibacteria*, which was referred to as candidate phyla radiation, were abundant in HKd, but were not found in HKm. OTUs of *Gallionellaceae* were not found in HKm and HKd, corresponding to a lower abundance of Fe-(hydro)oxides. *Cyanobacteria* accounted for 6.5% and 2.3% of the microbial community in HKs-Fe and HKd, respectively. HKs-Mn and HKm, which were not exposed to the surface, did not show OTUs of cyanobacteria. The genera *Pedobacter* sp., *Candidatus Kaiserbacteria* sp., and *Massilia* sp. were also found in the Mn-rich samples (HKd).

HKs-Mn, uniquely, contained a considerable proportion of anaerobic microorganisms (red in Figure 4). The class *Thermoanaerobaculia*, accounted for a large proportion (10.0% in Figure 4). The class of *Thermoanaerobaculia* includes two genera, namely *TPD-58* and *Thermoanaerobaculum*, in HKs-Mn. The following classes are, also, found: *Desulfomonilia* (3.1%), the phylum of *Desulfobacterota* (5.3%), and *Thermodesulfovibrionia* (1.6%). *Thermodesulfovibrionia* belongs to phylum *Nitrospirae*, which contained putative Mn-oxidizing bacteria [39].

### 3.3. Bacteria Associated with Mn Oxidation

The taxonomy of MAGs was determined, based on 120 concatenated single-copy bacterial genes (Table S4). Nine MAGs possessed Mn-oxidizing genes, indicating the presence of putative Mn-oxidizing bacteria (Table S5). In the HKs-Mn, MAGs of putative Mn-oxidizing bacteria, genus *Rhizobiaceae_RCIO01* (HKs107), previously reported as Mn-oxidizing bacteria [96], was successfully constructed.

In the HKm, MAGs of the putative Mn-oxidizing bacteria *Ramlibacter* sp. (HKm46) was constructed (Table S5). The genus *Ramlibacter* sp. Is, generally, an aerobic heterotroph and has not been recognized as a Mn-oxidizing bacteria. Other MAGs of putative Mn-oxidizing bacteria were assigned to thermophile, the class of *Blastocatellia* (HKm2), which, also, has not been reported to have Mn-oxidation capacity.

Sample HKd showed different characteristics. MAGs of the putative Mn-oxidizing bacteria, *Herminiimonas* sp. (HKm161) and *Hydrogenophaga* sp. (HKd102) were constructed (Table S5). *Herminiimonas* sp. has not been recognized as Mn-oxidizing bacteria, but *Hydrogenophaga* sp. was, previously, reported as Mn-oxidizing bacteria [46]. These are candidates for major Mn-oxidizing bacteria at each site. Beside those common Mn-oxidizing bacteria, some anaerobic bacteria were found to have Mn-oxidizing genes (see Section 3.5).

### 3.4. MCOs Utilization for Biological Mn Oxidation in Nature

Our metagenomic data indicated the prevalence of putative Mn-oxidizing genes encoding MCOs in the examined samples (Table S5). Among the nine MAGs in the present study, *moxA* (locus ID; CAJ19378) was the top hit, with an identity of approximately 70% of the HKs-Mn, HKm, and HKd (HKs107, HKm46, HKd161), respectively. Other putative Mn-oxidizing genes in HKs-Mn (HKs85, 166, 176, 177) were *moxA*, *mcoA* (locus ID; ABY98562), and *mnxG* (locus ID; PputGB1_2447), while those in HKm and HKd were *moxA* and *mcoA* (HKm2 and HKd102). These data confirm that MCOs were the dominant Mn-oxidizing genes around venting and downstream sites.

**Figure 5:**
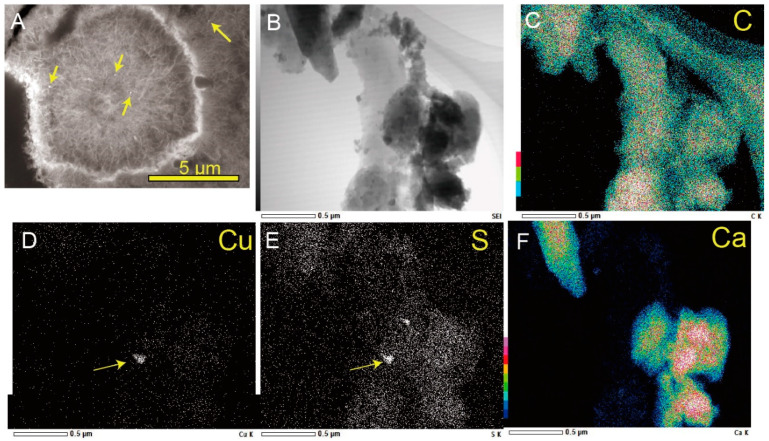
Cu-S nano-nuggets in MnO_2_/Organic complex. (**A**) FE-SEM image showing distribution of nanoscale Cu-S nuggets in MnO_2_/Organic complex structure (HKm). Yellow arrows indicate Cu- and S-bearing nuggets in organic matter. (**B**) TEM image showing Cu-S nuggets in organic matter and (**C**–**F**) distribution of S, Cu, C, and Ca, respectively. The spots of Cu are corresponded to those of S (yellow arrows in (**D**,**E**), but dark image in (**F**)).

Nanoscale textures and chemistry of organic matter in Mn-oxide spherules were analyzed, using high-resolution transmission electron microscopy (HR-TEM) and STEM. Cu-bearing nuggets (<300 nm, mostly 100–200 nm Figure 5A) have been, newly, found in the spherules. Such nuggets only occurred in organic matter in the spherules, and Mn oxides or carbonates in the same spherules never contained the Cu-bearing nuggets. Quantitative analyses by HR-TEM indicated that the nuggets were mostly made of Cu_x_S_y_ (Figure 5B–F), although the determination of specific stoichiometry was difficult. Cu-bearing nuggets are relevant for natural covellite (CuS) or chalcocite (Cu_2_S). Such Cu-bearing nuggets in organic matter have not been reported, previously, in terrestrial hot spring environments. The stability field of Cu_x_S_y_ was estimated, using the chemical data of HK hot spring water (Figure S3). The stability field was incompatible with the conditions of the samples, in which aragonite and goethite precipitate. These facts suggest that abiotic precipitation of Cu_x_S_y_ from hot spring water is not thermodynamically favored. In addition, FeS_2_ or FeS were not found in the examined samples, although hot spring water contains significant amounts of Fe^2+^. This suggests that Cu_x_S_y_ was not a simple product of microbial sulfate reduction (e.g., [97]). In nature, Mn oxides act as sponges to adsorb trace elements (e.g., [98]). Mn nodules or crusts on the modern ocean floor are known to abiotically accumulate Cu and other heavy metals, and they are comparable to several hundreds to thousands parts per million (ppm) (e.g., [99]). On the other hand, the Mn oxides in the examined samples did not show the enrichment of Cu (1.5 ppm) and other heavy metals (Table S8). Such observations suggest a unique mechanism to form Cu_x_S_y_ in the examined samples, rather than simple adsorption and enrichment on the surfaces of Mn oxides. We interpret this to mean that the novel Cu nuggets are traces of MCOs, after significant diagenetic modification from their original forms.

Nano-scale aggregations of biogenic metal sulfides within organic matter were reported, previously, from natural samples [100]. Metals in metal-binding proteins are bound with sulfur in amino acids, proteins, and polypeptides belonging to the sulfhydryl group. Nano-particles of metal sulfides were formed, after degradation of the original protein–metal compounds [97]. Similar nano-particles of various metal sulfides (e.g., Zn, Hg, Fe, Cd) have been found, in natural organic-rich samples. It is interpreted that Cu-binding proteins (MCOs) were degraded after cell death, and Cu and sulfur from organic molecules were trapped in non-permeable organic layers cemented in carbonates. H_2_S from deep sulfate reduction might join, as a part of sulfur, in this closed system. Aggregations were promoted by binding protein-rich organic matter with metal sulfides. In particular, cysteine stimulates large aggregations, up to ~100 nm diameter [100]. MCOs, generally, contain cysteines bound with Cu. Such high concentrations of Cu and organic molecules, including cysteine in closed systems, were responsible for Cu_x_S_y_ formation in the examined samples. Other organic sulfurs, also, contribute to form Cu sulfides. The finding of Cu_x_S_y_ is consistent with the detection of genes encoding MCOs in the same samples, supporting that MCOs were the major Mn-oxidizing genes in the venting area and downstream sites.

### 3.5. Role of Mn Oxidation in the Sinter Ecosystem

Phylogenetic analyses indicated that the microbial communities in Mn oxides differed at the sampling locality. Mn oxides in the venting site harbored a remarkable proportion of anaerobic microorganisms, such as sulfate-reducing bacteria (SRB) and Mn-reducing bacteria. In contrast, Mn oxides at the downstream harbored the aerobic heterotrophs. Putative Mn-oxidizing bacteria at the venting site were different from those at downstream sites.

The temperature of hot spring water was lower and more oxic at downstream sites, compared to the venting site. These factors are considerable reasons for the differences in the microbial community structures and Mn-oxidizing bacteria at each site (Figure 4, and Table S3). At both sites, biological Mn oxidation benefits the entire microbial community, and Mn oxides are utilized as electron acceptors. Alternatively, Mn oxides are utilized for the degradation and storage of organic matter in the microbial community [101,102].

Our analyses indicate that the following anaerobic bacteria have putative Mn-oxidizing genes: *Thermodesulfovibrionales* (HKs177), *Desulfobacterota* (HKs166), *Thermoanaerobaculia* (HKs85), and *Chloracidobacteriales* (HKs176) (Table S5)*. Desulfobacterota* and *Thermodesulfovibrionales* were SRB. Finding putative Mn-oxidizing genes in those anaerobic bacteria is enigmatic, and it is still uncertain whether those bacteria are actively oxidizing Mn(II) at the examined site.

Recent incubations of anaerobic phototrophs [40] and aerobic chemolithoautotrophs [39] showed microbial Mn(II) oxidation, with a help from other aerobic and anaerobic microbial communities. These studies indicate the importance of biogenic Mn oxides, for developing microbial communities at the interface of oxic and anoxic environments.

Yu and Leadbetter (2020) suggested that the class of putative Mn-oxidizing bacteria are phylogenetically closed to the phylum of *Nitropsirae*, which contains SRB classes. Our findings and previous results, further, imply that SRB, potentially, acquired the anaerobic Mn-oxidizing ability during its evolution, although conclusive evidence is, still, unavailable. Yu and Leadbetter (2020) proposed metabolic pathways for chemoautotrophic Mn oxidation and emphasized the postulation of Fe-S clusters with Cu-bearing protein, to transfer electrons in vitro. This model, further, implies the necessity of sulfur for Mn oxidation. The presence of SRB might be beneficial for Mn-oxidizing bacteria in the same microbial community, so that Mn-oxidizing bacteria could uptake essential sulfur species easily from SRB. This could be alternative explanation for detection of Mn oxides in SRB-bearing complexed microbial community. The presence of Mn oxides was also suggested to be beneficial to SRB for energy conservation (i.e., buttery) through metabolic electron transfers. This, further, implies that the inorganic Mn oxides are unified with microbial mats and have essential roles to sustain anaerobic microbial communities.

## 4. Conclusions

Geochemical, mineralogical, and metagenomic analyses were performed on Mn-oxide-rich sinters in Japan. Sub-micron scale spherical aggregates of Mn oxides were observed. HAADF analyses revealed that the Mn oxides were composed of poorly crystalline phyllomanganates, including δ-MnO_2_ (vernadite), hausmannite (Mn^2+^Mn^3+^_2_O_4_), and birnessite ((Na,Ca,K) × (Mn^4+^, Mn^3+^_2_)_2_O_4_·1.5H_2_O). Nanoscale layers of Mn oxides in each sphere were, often, intercalated with layers of organic matter, which are, rarely, found in marine Mn crust or nodules. The low crystallinities of the spherical Mn oxides and their close associations with organic matter support the biogenic origin of Mn oxides.

Several putative Mn-oxidizing genes encoding MCOs were identified, using metagenomic analyses. The predominant putative Mn-oxidizing genes were *moxA* and *mcoA*. Nanoscale nuggets of copper sulfides were, also, discovered in the layers of organic matter. Thermodynamic calculations indicated that conditions in the examined hot spring environment were not favorable for the abiotic precipitation of copper sulfides. In addition, other mineralogical and geochemical data excluded the possibility of the product, by microbial sulfate reduction or simple adsorption and enrichment on the surfaces of Mn oxides. Therefore, the novel copper sulfides are, most likely, degradation products of MCO-bearing proteins.

Enzymatically produced Mn oxides, most likely, acted as electron acceptors or helped in the degradation and storage of organic matter. These actions would help sustain and develop the overall aerobic and anaerobic microbial communities. Nine MAGs of putative Mn-oxidizing bacteria were detected. In particular, four of them appeared to be close associations of Mn-oxidizing genes with anaerobic bacteria, including SRB, although there was high uncertainty regarding whether anaerobic bacteria anaerobically oxidized Mn(II). The findings of the present study suggest that Mn oxides became a part of meso to thermophilic microbial mats and offer essential roles to sustain anaerobic microbial communities.

## Data Availability

The data that support the findings of this study are available from the corresponding author, Y.T.

## Supplementary Materials

The following supporting information can be downloaded at: https://www.mdpi.com/article/10.3390/life12060816/s1, Figure S1: (A) Electron microscopic image of the FIB-processed part (yellow area). (B,C) FIB-processed samples. The white square corresponds to (D). (D–I) and (K–Q) TEM images and chemical compositions of the FIB-processed Mn oxides. (J) Spectrum of area mapping in the field of view for (D). Cu was detected, Figure S2: Alignment of four conserved copper binding sites among putative Mn-oxidizing genes in nine MAGs in this study and known Mn-oxidizing genes, Figure S3: Eh–pH diagram of the Cu-S-Fe-O system with coexisting stability area for both aragonite and goethite, described by Geochemist Workbench Standard 12.0. Yellow represents the stability field of Cu_x_S_y_ (in this case covellite and chalcocite). Red represents the coexisting stability area for both aragonite and goethite. Blue indicates the pH conditions of the hot spring in HK. Calculations are conducted on the basis of the water chemistry of hot spring as follows: aCu = 10^−7.06^, aCa = 10^−1.796^, aHCO_3_ = 10^−1.678^, aNa^+^ = 10^−1.678^, aCl^−^ = 10^−1.602^, aMg^2+^ = 10^−2.301^, aSO4^2-^ = 10^−2.097^, aFe^2+^ = 10^−4.162^, aMn^2+^ = 10^−4.189^. (References [103,104,105,106,107,108,109,110,111,112,113,114,115,116,117,118] are cited in the supplementary materials).
