# Supplementary material for: Mineralogical and Genomic Constraints on the Origin of Microbial Mn Oxide Formation in Complexed Microbial Community at the Terrestrial Hot Spring
Source: Life (Basel). 2022 May 30;12(6):816. doi: 10.3390/life12060816 (PMC9224936; doi:10.3390/life12060816)
Supplement: Supplementary file 1 [file life-12-00816-s001.zip › life-1682017-supplementary/HK-Life-supplementary_materials.pdf]

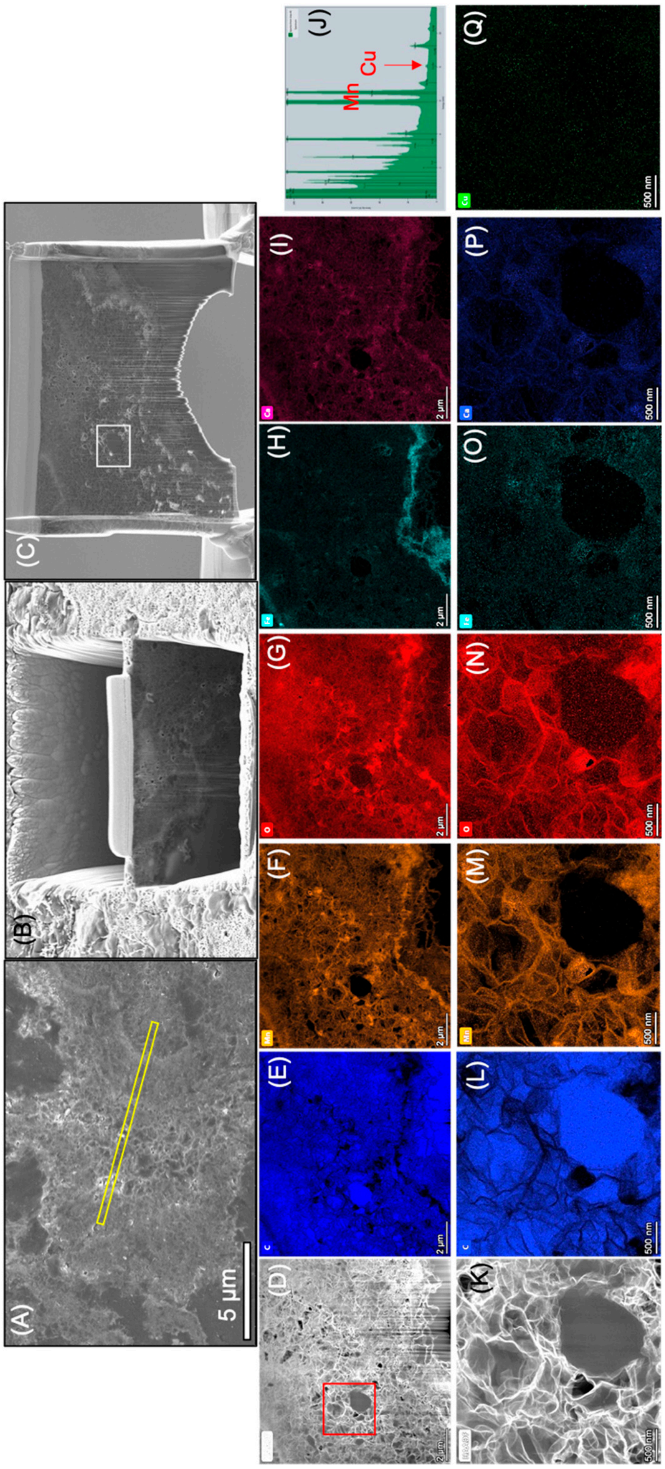

Figure S1 (A) Electron microscopic image of the FIB-processed part (yellow area). (B) and (C) FIB-processed samples. The white square corresponds to (D). (D)–(I) and (K)–(Q) TEM images and chemical compositions of the FIB-processed Mn oxides. (J) Spectrum of area mapping in the field of view for (D). Cu was detected.

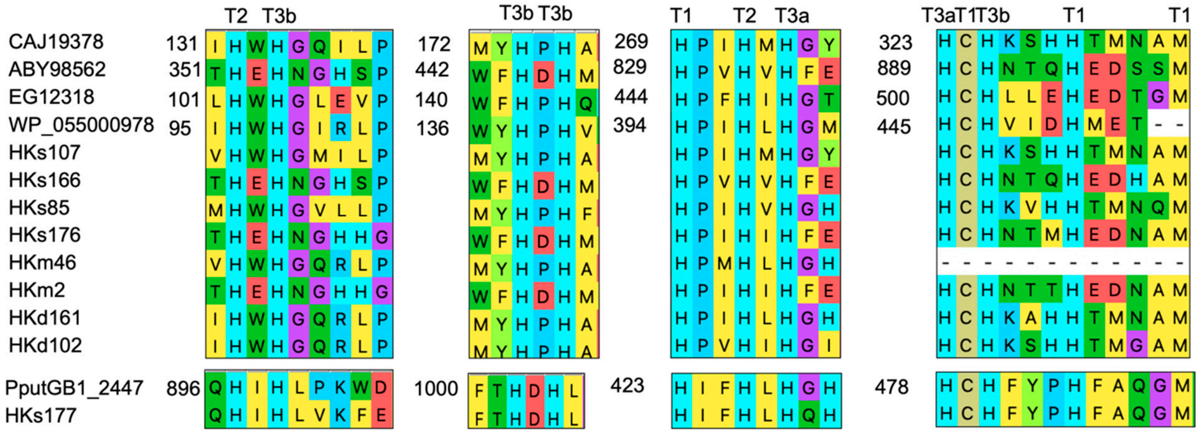

Figure S2 Alignment of four conserved copper binding sites among putative Mn-oxidizing genes in nine MAGs in this study and known Mn-oxidizing genes.

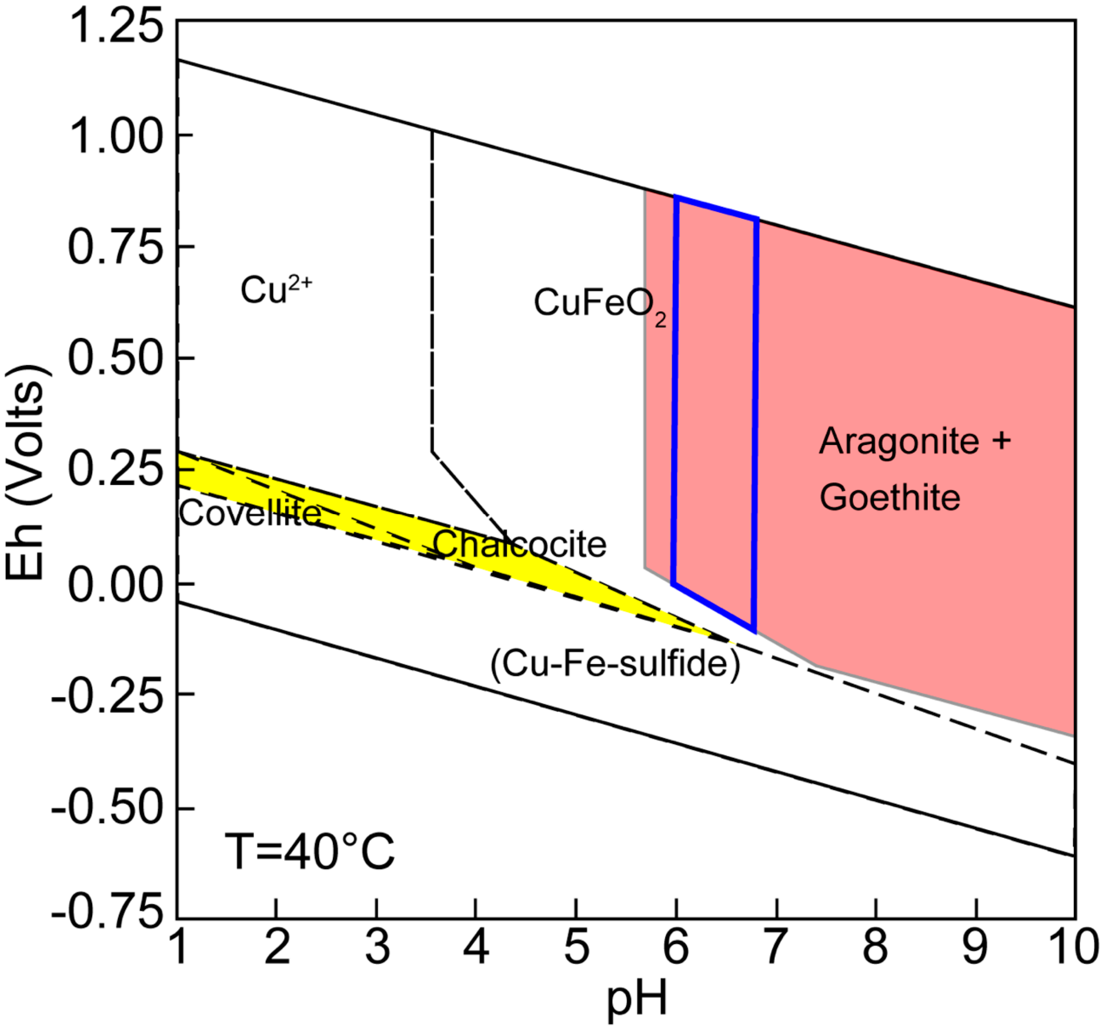

Figure S3 Eh–pH diagram of the Cu-S-Fe-O system with coexisting stability area for both aragonite and goethite, described by Geochemist Workbench Standard 12.0. Yellow represents the stability field of Cu_xS_y (in this case covellite and chalcocite). Red represents the coexisting stability area for both aragonite and goethite. Blue indicates the pH conditions of the hot spring in HK. Calculations are conducted on the basis of the water chemistry of hot spring as follows: aCu=$10^{-7.06}$, aCa=$10^{-1.796}$, aHCO$_3$=$10^{-1.678}$, aNa$^+$=$10^{-1.678}$, aCl$^-$=$10^{-1.602}$, aMg^{2+}=$10^{-2.301}$, aSO4^{2-}=$10^{-2.097}$, aFe^{2+}=$10^{-4.162}$, aMn^{2+}=$10^{-4.189}$.
